# Supplementary material for: An Intrinsic Host Defense against HSV-1 Relies on the Activation of Xenophagy with the Active Clearance of Autophagic Receptors
Source: Cells. 2024 Jul 26;13(15):1256. doi: 10.3390/cells13151256 (PMC11311385; doi:10.3390/cells13151256)
Supplement: Supplementary file 1 [file cells-13-01256-s001.zip › Pino-Belmar et al., 2024_CELLS_Supplementary Materials.pdf]

*Supplementary Materials*

# An intrinsic host defense against HSV-1 relies on the activation of xenophagy with the active clearance of autophagic receptors

**Pino-Belmar C., et al.**

**Table S1. Primer pairs sequences used for RT-qPCR analyses.**

**Figure S1. HSV-1 infection reduces the levels of autophagic receptors to a high multiplicity of infection.** H4 cells uninfected (M; Mock) and infected with HSV-1 Strain F with different viral loads at MOI 0.1, 1, 5 and 10 were analyzed at 8 h post-infection (hpi). (**A**) Protein extracts were subjected to immunoblot using monoclonal antibodies against, SQSTM1/p62, OPTN1, NBR1 and NDP52. Representative proteins of the replicative cycle of HSV-1 were detected with monoclonal antibodies: ICP27 (immediate early protein), ICP8 (early protein), and ICP5 (late protein) to control infection. β-ACTIN was used as a loading control. Positions of molecular weight markers (kDa) are indicated on the left.A representative image from immunoblot was displayed in High Exposure (High Exp) to indicate modified SQSTM1/p62 species, in comparison to the image captured at Low Exposure (Low Exp). Densitometric quantification of (**B**) SQSTM1/p62, (**C**) OPTN1, (**D**) NBR1 and (**E**) NDP52 protein levels were normalized to β-ACTIN. Statistical significance was determined by One-Way ANOVA, followed by Tukey's Test. The bars represent the mean ± SD of biological replicates (SQSTM1/p62 $n$=3; OPTN1 $n$=3; NBR1 $n$=3; NDP52 $n$=3) n.s., not significant, *$p$<0.05, **$p$<0.01.

**Figure S2. Transcriptional regulation of autophagic receptors during HSV-1 infection.** Either HaCaT cells uninfected (Mock) and infected cells (**A**-**D**) or H4 cells uninfected (Mock) and infected cells (**E**-**H**) with HSV-1 Strain F at MOI 10 were analyzed at 4, 8, 18 and 24 h post-infection (hpi). mRNA levels of (**A**-**E**) *P62* (SQSTM1/p62) (**B**-**F**) *OPTN* (OPTN1), (**C**-**G**) *NBR1* y (**D**-**H**) *CALCOCO2* (NDP52) were measured using RT-qPCR from both cell types. All data were normalized for *GADPH* expression. Statistical significance was determined by One-Way ANOVA, followed by Tukey's Test. Bars represent means ± SD of biological replicates (*P62* $n$=3; *OPTN* $n$=3; *NBR1* $n$=3; *CALCOCO2* $n$=3) n.s., not significant, *$p$<0.05, **$p$<0.01, ***$p$<0.001.

**Figure S3. EBSS induces autophagy, a process enhanced during HSV-1 infection.** (**A**) H4 cells were were cultured in medium full supplemented for 8 hours (Control) or in Earle's balanced salts solution (EBSS) for the indicated time to induce autophagy and 0.1 mM Chloroquine (CQ) was used as autophagic flux inhibitor for 4 h. Protein extracts were subjected to immunoblot using a polyclonal antibody against LC3. β-ACTIN was used as a loading control. Positions of molecular weight markers (kDa) are indicated on the left. Densitometric quantification of (**B**) LC3-I and (**C**) LC3-II protein levels were normalized to β-ACTIN. Statistical significance was determined by Student's T-test. Bars represent the mean ± SD of biological replicates (LC3-I $n$=3; LC3-II $n$=3) n.s., not significant; *$p$<0.05, ****$p$<0.0001. (**D**) H4 uninfected and infected with HSV-1 Strain F at MOI 10, were cultured in medium-full supplemented for 8 h (Control) or in Earle's balanced salts solution (EBSS) for 4 or 8 h to induce autophagy. Protein extracts were subjected to immunoblot (Figure 3). LC3 levels were assayed with a polyclonal antibody against LC3 that recognizes LC3-I and LC3-II. The cytosolic LC3 ratio was expressed as LC3-II/LC3-I in response to lack of nutrients in uninfected and HSV-1 infected cells. Statistical significance was determined by One-Way ANOVA, followed by Tukey's test. The bars represent the mean ± SD of biological replicates (LC3-I n=4; LC3-II n=4) *p<0.05, **p<0.01, ***p<0.001, ****p<0.0001.

**Figure S4. Effect of proteasome inhibitor MG132 on the levels of autophagic receptors during HSV-1 infection.** H4 cells uninfected (Mock) and infected with HSV-1 Strain F at MOI 10, were treated with either vehicle DMSO (Control) or 20 µM MG132 for 8 h to inhibit proteasomal degradation. Protein extracts were subjected to immunoblot using monoclonal antibodies against (**A**) SQSTM1/p62, OPTN1, NBR and NDP52. HSV-1 ICP8, an early was used to control the infection. β-ACTIN was used as a loading control. Positions of molecular weight markers (kDa) are indicated on the left. Densitometric quantification of (**B** and **C**) SQSTM1/p62, (**D** and **E**) OPTN1, (**F** and **G**) NBR1, and (**H** and **I**) NDP52 protein levels were normalized to β-ACTIN. Statistical significance was determined by Student's T-test. Bars represent means ± SD of biological replicates (SQSTM1/p62 $n$=3; OPTN1 $n$=3; NBR1 $n$=3; NDP52 $n$=3) n.s., not significant, **$p$<0.01.

**Figure S5. The role of US11 HSV-1 protein is not associated with the regulation of LC3 levels.** Uninfected (M; Mock) and H4 cells infected with: HSV-1 Strain F (WT), HSV-1 strain R3616 *null* mutant for ICP34.5 (Δ34.5), and HSV-1 strain R3631 *null* mutant for US11 (ΔUS11) at MOI 10 for 8 h. Protein extracts were subjected to immunoblot. (**A**) LC3 levels were assayed with a polyclonal antibody against LC3 that recognizes LC3-I and LC3-II. β-ACTIN was used as a loading control. Positions of molecular weight markers (kDa) are indicated on the left. Densitometric quantification of (**B**) LC3-I and (**C**) LC3-II protein levels were normalized to β-ACTIN. Statistical significance was determined by One-Way ANOVA, followed by Tukey's Test. Bars represent means ± SD of biological replicates (LC3-I $n$=3; LC3-II $n$=3) n.s., not significant, *$p$<0.05, **$p$<0.01, ***$p$<0.001.

**Figure S6. Cytopathic effect induced by HSV-1 strain F and mutant virus.** Monolayers of H4 cells uninfected (Mock) and infected with: HSV-1 Strain F (WT), HSV-1 strain R3616 *null* mutant for ICP34.5 (Δ34.5), and HSV-1 strain R3631 *null* mutant for US11 (ΔUS11) at MOI 10 were visualized 8 h post-infection (hpi) by bright-field microscopy. Scale bar, 50 μm.
