# Supplementary material for: An Intrinsic Host Defense against HSV-1 Relies on the Activation of Xenophagy with the Active Clearance of Autophagic Receptors
Source: Cells. 2024 Jul 26;13(15):1256. doi: 10.3390/cells13151256 (PMC11311385; doi:10.3390/cells13151256)
Supplement: Supplementary file 1 [file cells-13-01256-s001.zip › Table S1.pdf]

Primer pairs sequences used for RT-qPCR analyses

Target	Oligonucleotide Primer Sequences
GADPH	Fw: 5'-TGGAAGGACTCATGACCACAG-3'<br>Rv: 5'-CAGCTCAGGGATGACCTTGC-3'
P62 **(SQSTM1/p62)**	Fw: 5'-AATCAGCTTCTGGTCCATCG-3'<br>Rv: 5'-TTCTTTTCCCTCCGTGCTC-3'
NBR1	Fw: 5'-ATCCAGTGGATAGATTGGAGACCTCCG-3'<br>Rv: 5'-CAATGCAAGGACCGTCCGCTA-3'
OPTN **(OPTN1)**	Fw: 5'-TCCAGGTGACATCTCTGTTT-3'<br>Rv: 5'-CCTCTGTTTTAGCCTGTTCC-3'
CALCOCO2 **(NDP52)**	Fw: 5'-CTACCATGGAGGAGACCATC-3'<br>Rv: 5'-CCCACCTGCAATTTCTGTC-3'
**UL29 (HSV-1 ICP8)**	Fw: 5'-CATCAGCTGCTCCACCTCGCG-3'<br>Rv: 5'-GCAGTACGTGGACCAGGCGGT-3'

Table S1
