# Supplementary figures and images for: An Intrinsic Host Defense against HSV-1 Relies on the Activation of Xenophagy with the Active Clearance of Autophagic Receptors
Source: Cells. 2024 Jul 26;13(15):1256. doi: 10.3390/cells13151256 (PMC11311385; doi:10.3390/cells13151256)

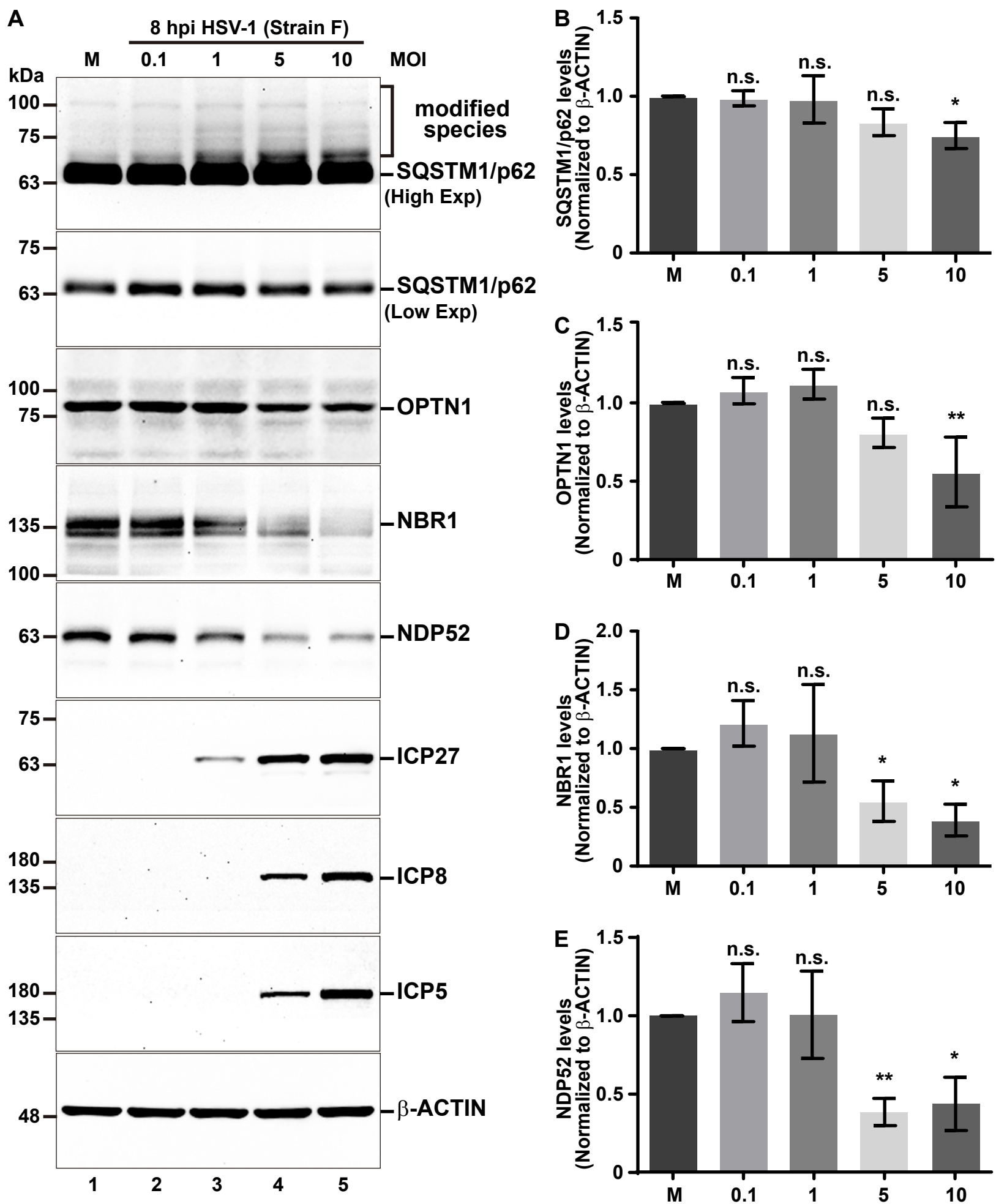

Figure S1

Supplement: Supplementary file 1 [file cells-13-01256-s001.zip › Figure S1.pdf]

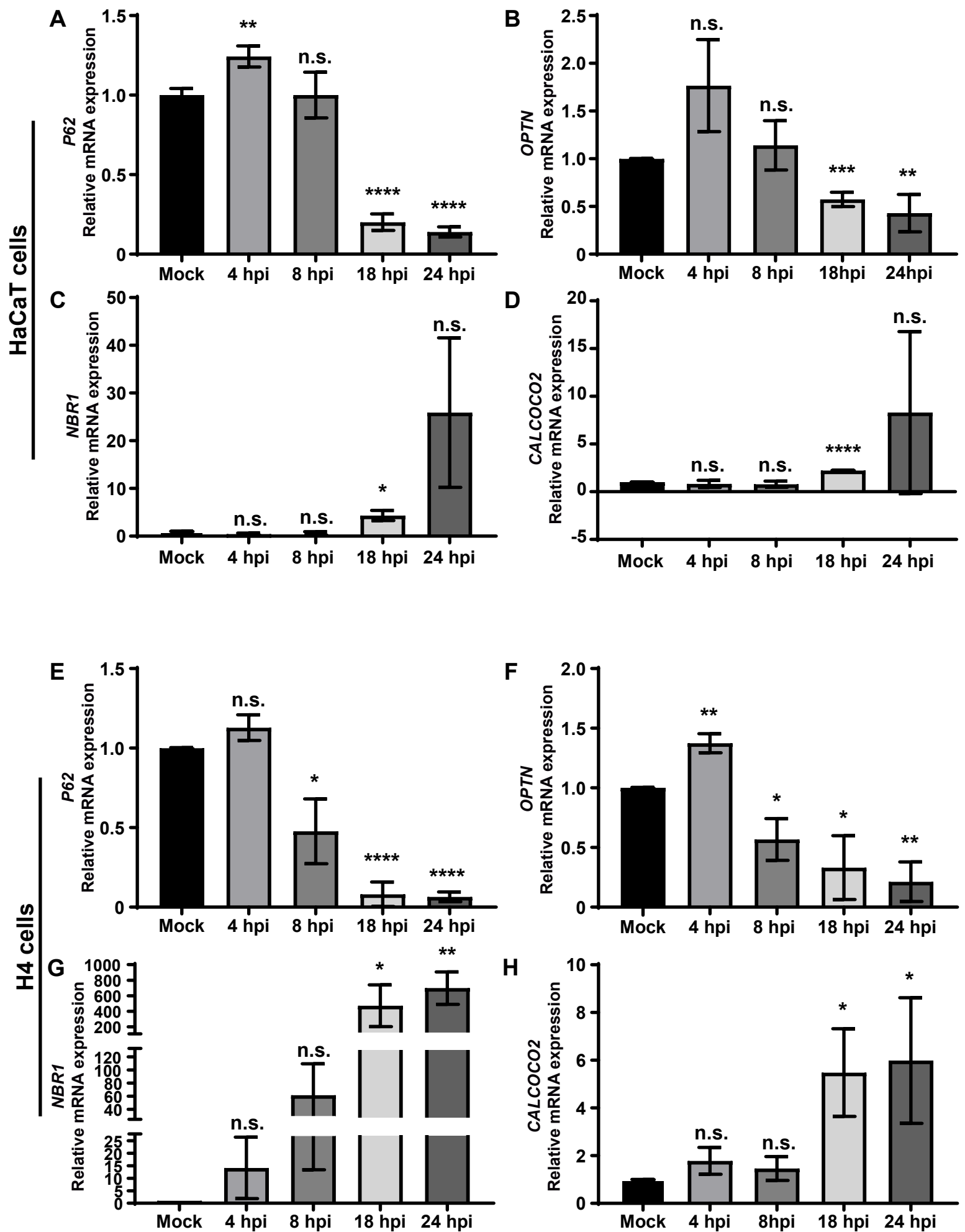

**Figure S2**

Supplement: Supplementary file 1 [file cells-13-01256-s001.zip › Figure S2.pdf]

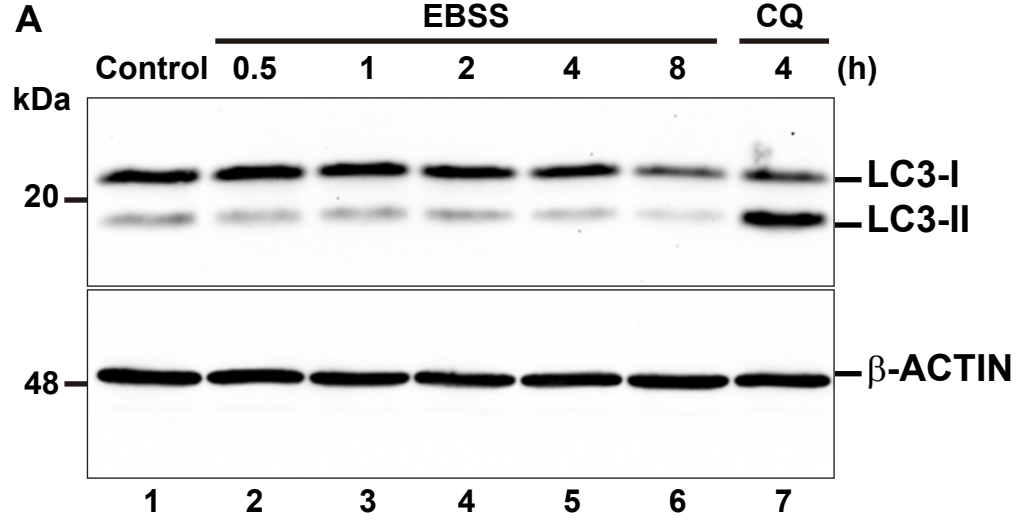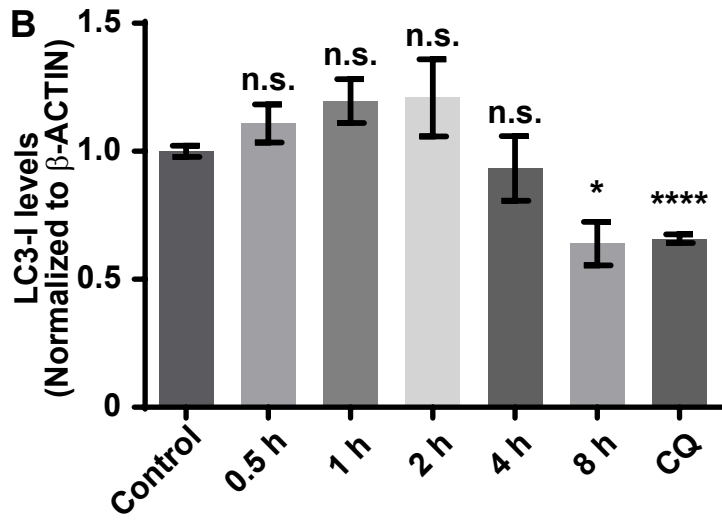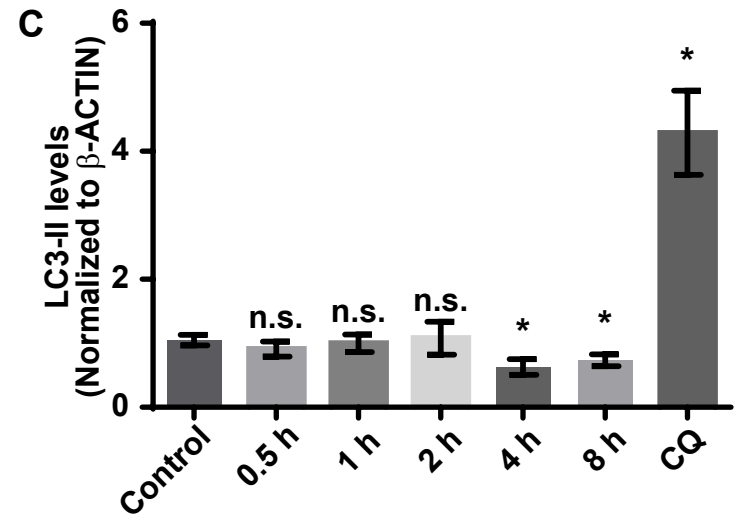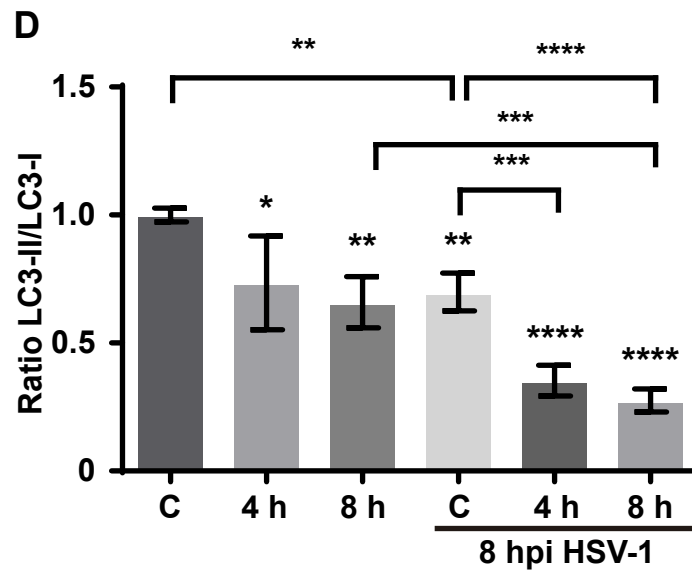

**Figure S3**

Supplement: Supplementary file 1 [file cells-13-01256-s001.zip › Figure S3.pdf]

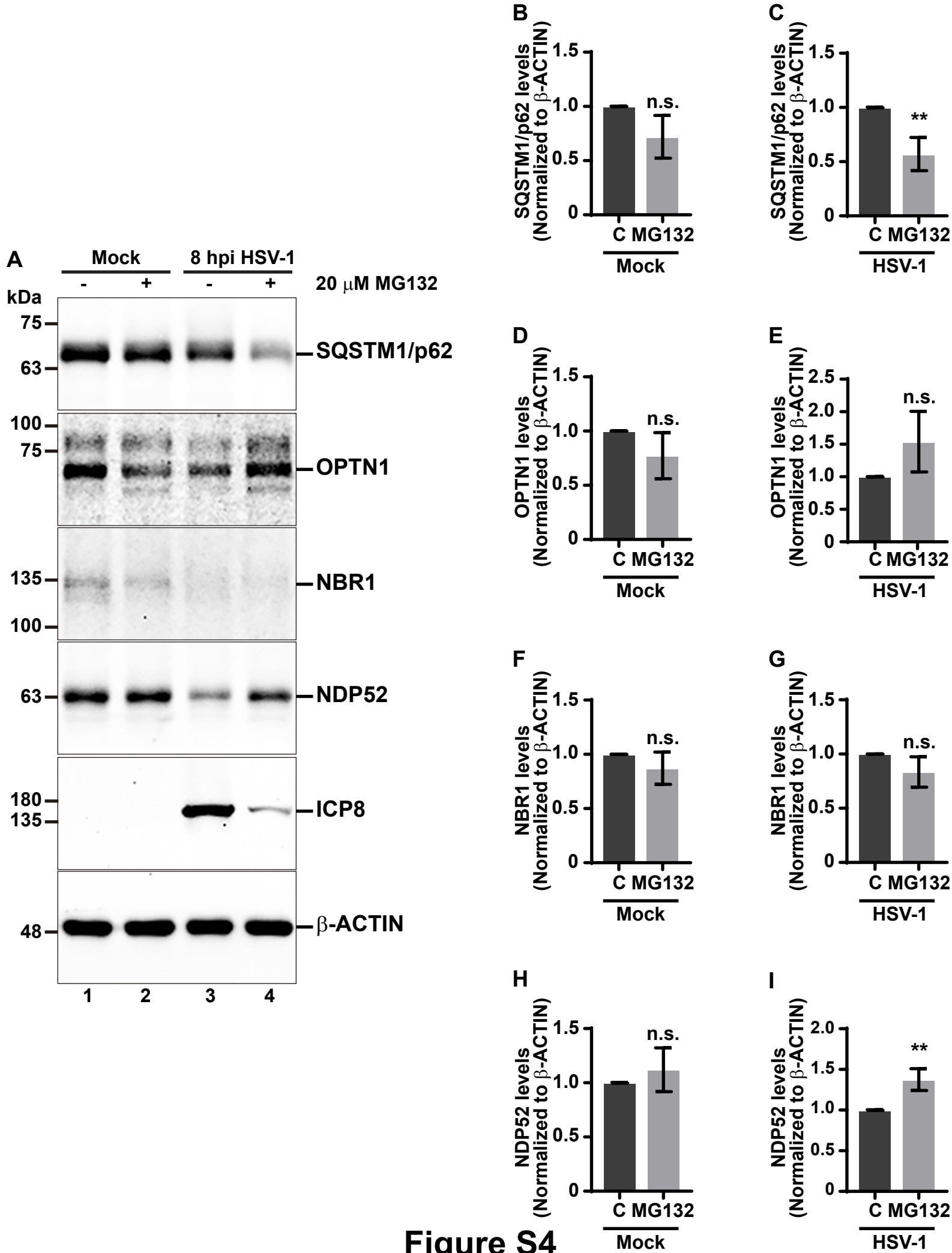

**Figure S4**

Supplement: Supplementary file 1 [file cells-13-01256-s001.zip › Figure S4.pdf]

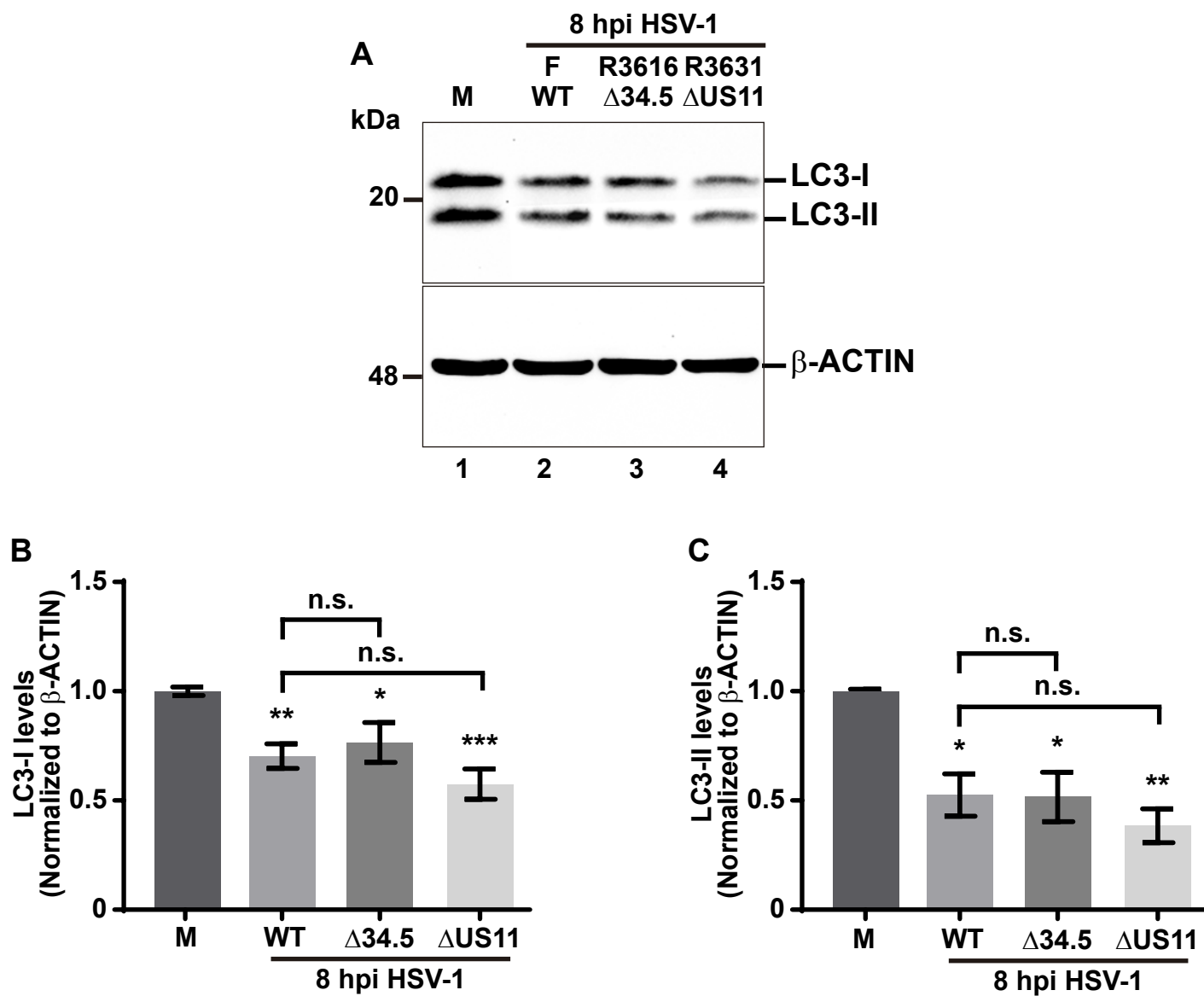

**Figure S5**

Supplement: Supplementary file 1 [file cells-13-01256-s001.zip › Figure S5.pdf]

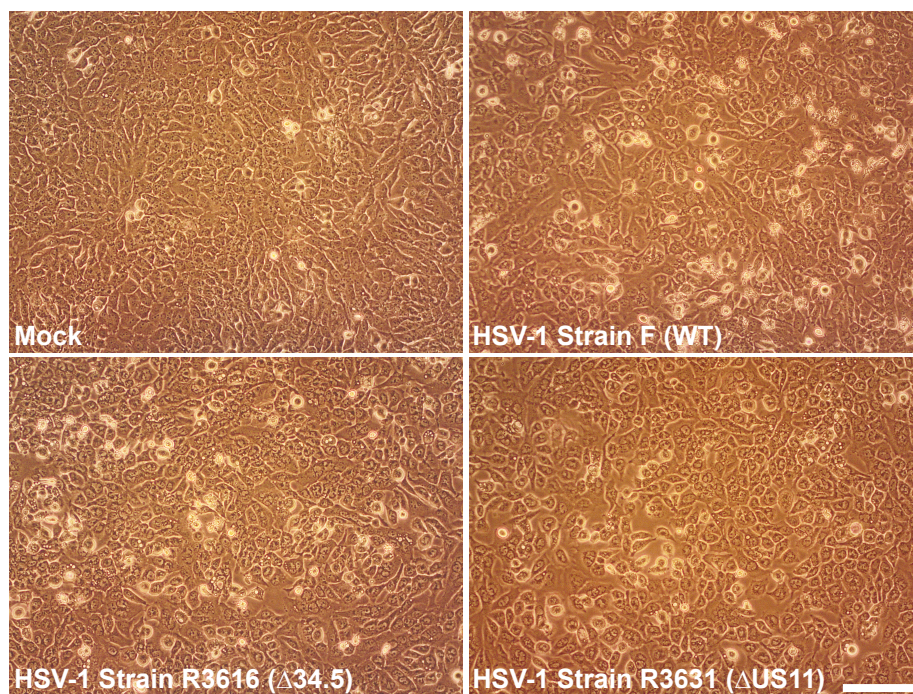

**Figure S6**

Supplement: Supplementary file 1 [file cells-13-01256-s001.zip › Figure S6.pdf]
